# Using machine learning to detect coronaviruses potentially infectious to humans

**DOI:** 10.1038/s41598-023-35861-7

**Published:** 2023-06-08

**Authors:** Georgina Gonzalez-Isunza, M. Zaki Jawaid, Pengyu Liu, Daniel L. Cox, Mariel Vazquez, Javier Arsuaga

**Affiliations:** 1 Department of Microbiology and Molecular Genetics, University of California, Davis, CA USA; 2 Department of Molecular and Cellular Biology, University of California, Davis, CA USA; 3 Department of Mathematics, University of California, Davis, CA USA; 4 Department of Physics, University of California, Davis, USA

**Keywords:** Computational biology and bioinformatics, Structural biology

## Abstract

Establishing the host range for novel viruses remains a challenge. Here, we address the challenge of identifying non-human animal coronaviruses that may infect humans by creating an artificial neural network model that learns from spike protein sequences of alpha and beta coronaviruses and their binding annotation to their host receptor. The proposed method produces a human-Binding Potential (h-BiP) score that distinguishes, with high accuracy, the binding potential among coronaviruses. Three viruses, previously unknown to bind human receptors, were identified: Bat coronavirus BtCoV/133/2005 and Pipistrellus abramus bat coronavirus HKU5-related (both MERS related viruses), and *Rhinolophus affinis* coronavirus isolate LYRa3 (a SARS related virus). We further analyze the binding properties of BtCoV/133/2005 and LYRa3 using molecular dynamics. To test whether this model can be used for surveillance of novel coronaviruses, we re-trained the model on a set that excludes SARS-CoV-2 and all viral sequences released after the SARS-CoV-2 was published. The results predict the binding of SARS-CoV-2 with a human receptor, indicating that machine learning methods are an excellent tool for the prediction of host expansion events.

## Introduction

Most novel viral human diseases, particularly those that have caused recent epidemics, are known to have originated in non-human animal hosts^1–3^. Host expansion, the ability of a virus to cross species, is a key step in the evolution of such viruses^3–5^. COVID-19 is a recent example of a disease caused by a host expansion event that permitted SARS-CoV-2, a SARS-related coronavirus, to propagate from a yet unknown non-human animal to humans^5^. Alpha and beta coronaviruses affect a wide range of animals interacting with humans, including farm animals and camels, thus facilitating zoonotic transmission^6, 7^. Moreover, all seven human coronaviruses belong to either the alpha or beta coronavirus genus^7^. While several studies have confirmed bats and rodents as natural hosts for the alpha and beta coronaviruses affecting humans, there is evidence of intermediate hosts that facilitate evolutionary events, leading to strains that eventually propagate in humans^1, 6, 8^. Determining which non-human animal viruses may infect humans remains a challenge.

Experimental evidence is still the gold standard used to determine whether a virus can infect a host^9, 10^. However, the complete host range of a virus is often unknown. Recent studies have used diverse in-silico techniques to predict viral hosts and host expansion events, including qualitative expert analysis^11^, probabilistic^12^ and machine learning (ML)^13–17^models.

The problem of host prediction is commonly addressed using similarity analysis of viral genomes, where similar genomes are more likely to share the same hosts^10, 18^. Host prediction through genome similarity can be achieved by alignment-based or alignment-free approaches^17, 19^. Computational efficiency of alignment-based approaches decreases with the product of the lengths of the sequences being aligned^19, 20^ and are sensitive to genome rearrangements^19–21^. These observations suggest alignment-free approaches may be preferred when datasets are very large or sequences in the dataset are the product of recombination events. However, most alignment-free approaches disregard the relative position of the residues along the sequence^14^.

Some alignment-free studies aimed at predicting the host of a specific species of virus^13, 14^, while others^15–17^ created models to uncover signals common to different viruses (*e.g.* Zika, influenza, coronavirus) affecting a large group of hosts such as Chordata (vertebrates and others)^15, 17^. Although common signals between completely different families of viruses are useful for host prediction, these studies include only a limited number of representatives of each taxa across hosts and disregard the specific properties of the virus, preventing further mechanistic analysis of host expansion pathways.

In this work, we study the potential of alpha and beta coronaviruses to cause human infection. In particular, we aim at predicting whether the spike (S) protein of a coronavirus binds a human receptor. The S protein decorates the exterior of the viral envelope and is key in host expansion since its binding to the host receptor protein triggers the infection process^22–24^. Starting with a collection of amino acid sequences from the S protein, we build a machine learning model that predicts binding to a human host receptor. We propose a skip-gram model which uses a neural network to transform the sequences into vectors. These vectors encode the relationship between neighboring protein sequences of length k (*i.e.* k-mers). A classifier uses these vectors to score each sequence according to its binding potential to a human receptor. We call this score the human-binding potential (h-BiP). We use a dataset consisting of 2,534 unique spike sequences from alpha and beta coronaviruses spanning all clades and variants (see “Methods”). The classifier is highly accurate, and its h-BiP score is highly correlated with sequence identity against human viruses. Moreover, the proposed h-BiP score also discriminates the binding potential in cases with similar sequence identity and detects binding in cases of low sequence identity. We identify three viruses, Bt133^25^, Pipistrellus abramus bat coronavirus HKU5-related (HKU5r)^26^ and LYRa3^27^, with high h-BiP values and yet unknown human binding properties. Consistent with this finding, a phylogenetic analysis shows that Bt133, HKU5r and LyRa3 are related to non-human viruses known to bind human receptors. Furthermore, a multiple sequence alignment of the receptor binding motifs (RBM) of Bt133 and of LYRa3 with their related viruses revealed that they conserve the contact residues with the human receptor. Molecular dynamics (MD) of the receptor binding domain (RBD) validates binding and identifies contact residues with human receptors. Finally, we test whether this model can be used for the surveillance of host expansion events. We emulate the conditions prior to SARS-CoV-2 emergence by excluding from the training set all coronavirus sequences published after December 31^st^, 2019 and find that the re-trained model predicts binding of the wild type of SARS-CoV-2 to a human receptor.

## Results

### h-BiP: a machine learning approach for scoring human-binding potential of coronavirus sequences

**Figure 1:**
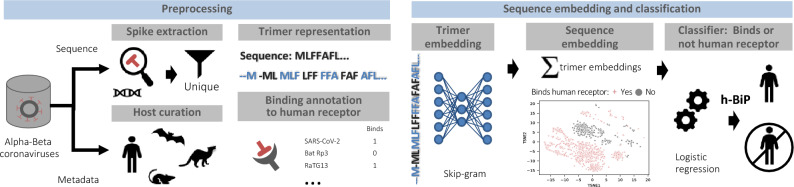
Methodological workflow of the human Binding Potential (h-BiP) score. Left: preprocessing sequences from alpha and beta coronaviruses. Top: whether the S protein was available from annotation or by extraction from whole-genome, the dataset consists of 2534 unique S protein sequences. Each protein sequence is transformed into a trimer (3 amino acid) representation by sliding a window one amino acid at a time. Bottom: we curated the host field and annotated the sequences according to their binding status to human receptors. Regardless of the host, a virus is considered positive for binding if there is experimental evidence of binding to a human receptor. Right: a skip-gram model uses a neural network to generate trimer embeddings of a fixed dimension (d = 100). These trimer embeddings are numerical vectors that encode information from all neighboring trimers within a context window in the protein sequence. Next, we compute the final sequence embedding (d = 100) by adding up all of its trimer embeddings. The scatterplot shows a visualization for the embeddings from all viruses after using t-distributed stochastic neighbor embedding (tsne) to reduce dimensionality. Finally, all sequence embeddings feed a classifier (logistic regression) to produce the h-BiP score that learns from the binding information of alpha and beta coronaviruses. An h-BiP score greater than or equal to 0.5 flags the virus as likely for human binding.

We propose a human-Binding Potential (h-BiP) score that assigns a value between 0 and 1 to spike proteins of alpha and beta coronaviruses. The outline of the method to obtain h-BiP is shown in Fig. 1, and a full description is available in “Methods” section. First, we partitioned each protein sequence as a sequence of trimers, which we used to produce trimer embeddings in a high-dimensional Euclidian space. We selected a skip-gram model^28^ to ensure that each trimer embedding was informed by neighboring trimers within a context window. Next, an embedding for the entire protein sequence was generated by adding all of its trimer embeddings. Each coronavirus, together with all its available variants in the dataset, was labeled as positive or negative according to its published binding annotation to any, known or unknown, human receptor (Table 1). To produce the h-BiP score, we built a logistic regression classifier on the sequence embeddings to predict binding. Viruses with h-BiP score of 0.5 or higher were classified as likely to bind a human receptor.

**Table 1:** Viruses with evidence of binding to a human receptor.

Alpha and beta coranaviruses that bind to a human receptor
RaTG13^29, 30^, LYRa11^29, 31^, WIV1^8, 29, 32^, WIV16^8, 32^, RsSHC014^29, 31^, Rs4231^31^, Rs4084^31, 33^, Rs4874^34^, Rs7327^34^, Rs4231^34^, Khosta-1^35^, Khosta-2^35^, Ty-HKU4^24, 36^, PCoV^30^, A022G^32^, SZ3^32^, B039G^32^, DcCoV HKU23^37^, BCov isolate Alpaca^44^, HKU25^36^, YN2018B^33^, Rs9401^33^, Rs3367^33^, Rs4081^38^, As6526^38^, Rs4237^38^, Shaanxi 2011^38^, Yunnan 2011^38^, HKU3-13^38^, NeoCoV^39^, HKU5^40^, PDF2180^39^
Human coronaviruses found in non-humans hosts
SARS-CoV-2 in multiple animals, hCov-229E: camel alphacoronavirus, hCoV-OC43 in chimpanzee, MERS Middle East strain in camelids^41^, hCov-229E: 229E-related bat CoV, hCov-229E: Alpaca respiratory CoV, SARS-CoV in minks, SARS-CoV-2 in minks, SARS-CoV civet, MERS African strain in camelids^42^
Human coronaviruses found in humans hosts
HEC, hCoV-229E, hCoV-HKU1, hCoV-NL63, hCoV-OC43, MERS, SARS-CoV, SARS-CoV-2

Dataset includes all variants from each of these coronaviruses.

### The h-BiP score is highly accurate in predicting binding to human receptors for alpha and beta coronaviruses

We split the data into training (85%) and testing sets (15%). Human coronaviruses account for 61% of the dataset and 50% of the non-human coronaviruses correspond to Porcine epidemic diarrhea virus. To ensure that training and test dataset have a similar composition we stratified by the following groups: hCoV-OC43, hCoV-HKU1, MERS, SARS-CoV-1, SARS-CoV-2, hCoV-NL63, hCoV-229E, other MERS-related, other Sarbecovirus, other Betacoronavirus, porcine epidemic diarrhea virus, other Alphacoronavirus.

Table 2 shows the prediction results for the full data set. The method achieved 99% accuracy, 99% sensitivity and 99% specificity in the test data set (see Supplementary Fig. S1). A total of 805 sequences had an h-BiP score less than 0.5. Of these, 796 were true negatives. While binding status was unknown for most of these sequences, viruses such as BM48-31 and Rf1 for which experimental studies found no evidence of binding to a human receptor^38^ were confirmed as non-binding by h-BiP (i.e. h-BiP score < 0.5). Three viruses with unknown binding status had an h-BiP score ≥ 0.5, suggesting they may potentially bind human receptors. Bat coronavirus BtCoV/133/2005 (Bt133), Pipistrellus abramus bat coronavirus HKU5-related (HKU5r) and Rhinolophus affinis coronavirus isolate LYRa3. Nine viruses were classified as false negatives (see Table 3). The h-BiP scores of these viruses ranged from 0.13 to 0.44.

**Table 2:** Confusion matrix for h-BiP on alpha and beta coronaviruses.

h-BiP	Binds to human receptor		Total
	Negative/unknown	Positive	
Negative (h-BiP < 0.5)	TN = 796	FN = 9	805
Positive (h-BiP ≥ 0.5)	FP = 3 (unknown)	TP = 1726	1729
Total	799	1735	2534

*TN* true negatives, *FN* false negatives, *FP* false positives, *TP* true positives.

**Table 3:** Viruses known to bind human receptors for which h-BiP is smaller than 0.5 (false negatives).

Accession	Virus	Human receptor	h-BiP score
JX993987	Bat coronavirus Rp/Shaanxi2011		0.163
KY417142	Bat SARS-like coronavirus isolate As6526		0.146
KY417147	Bat SARS-like coronavirus isolate Rs4237		0.221
GQ153548	Bat SARS coronavirus HKU3-13		0.208
KY417143	Bat SARS-like coronavirus isolate Rs4081		0.129
MZ190138	Bat SARS-like coronavirus Khosta-2	ACE2	0.444
ACJ35486	Human enteric coronavirus 4408	9–0-acetylated sialic acid	0.244
ACT11030	Human enteric coronavirus 4408	9–0-acetylated sialic acid	0.261
ABI93999	Bovine coronavirus isolate Alpaca	N-acetyl-9-O-acetylneuraminic acid	0.176

### The h-BiP score is consistent with sequence identity and expands viral classification

**Figure 2:**
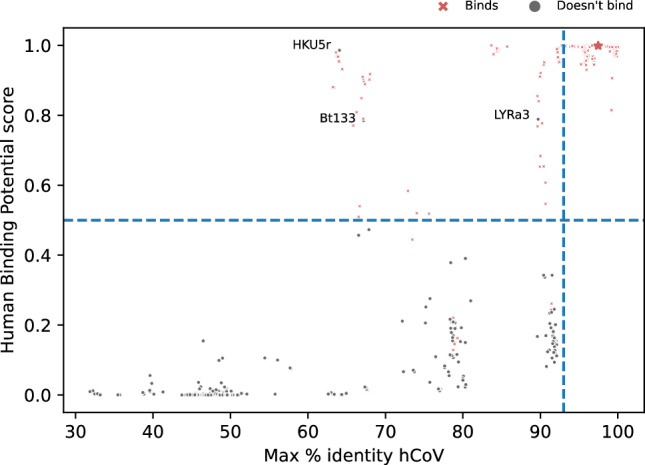
Comparison of sequence % identity and h-BiP score for alpha and beta coronaviruses. The x-axis represents the maximum % identity computed from a particular virus against the seven known human coronaviruses. The y-axis shows the h-BiP score. Each point in the graph represents a sequence in the dataset. Regardless of their host, red crosses depict sequences of viruses known to bind a human receptor, and grey points represent those viruses not known to do so. Points above the blue dashed horizontal line have a h-BiP score greater or equal than 0.5 (i.e. positive for binding). The blue dashed vertical line is the 97% identity reference line. The spike protein of bat coronavirus RaTG13 (depicted with a red star) is known to bind to human receptor hACE2 and it has a 97.46% amino acid identity against SARS-CoV-2 and a 0.999 h-BiP score. Three viruses with h-BiP ≥ 0.5 and yet unknown binding, Bt133, HKU5r and LYRa3 are highlighted.

We verified that the proposed model, while consistent with sequence alignment results, provides additional information. Percent sequence identity (% identity) of a newly detected virus with known human viruses^10, 18^ is often used to assess human infectivity. We computed the pairwise % identity between each of the 7 human coronaviruses and the S protein sequences in our dataset and selected the maximum for each sequence. All cases with 93 or higher protein % identity with known human coronaviruses had a h-BiP score greater than 0.5. Furthermore, the Pearson correlation between these maximum % identities and our proposed h-BiP score was 0.96 (Fig. 2). Hence, we conclude that our method is consistent with standard sequence identity approaches.

Our study identified 26 bat viruses with maximum % identity < 83% and an h-BiP ≥ 0.5. There is experimental evidence that these viruses bind to a human receptor, except for Bt133 and HKU5r (Table 1). A protein BLAST^43^ against all coronaviruses confirmed that Bt133 and HKU5r have a low % identity with any human coronavirus. The h-BiP score also discriminates between viruses that have similar % identity. For instance, the spike proteins from bovine and pangolin coronaviruses have % identities that range from 90 to 92% (see Fig. 2). The h-BiP scores for pangolin coronaviruses and bovine coronaviruses are greater than 0.97 and less than 0.4, respectively. This finding agrees with current experimental results that show binding of pangolin coronaviruses to human receptors^30^. To our knowledge, only the alpaca isolate from Bovine coronaviruses^44^ has been reported for human infections.

### The h-BiP score predicts that the S protein of viruses Bt133, HKU5r and LYRa3 bind to human receptors

**Figure 3:**
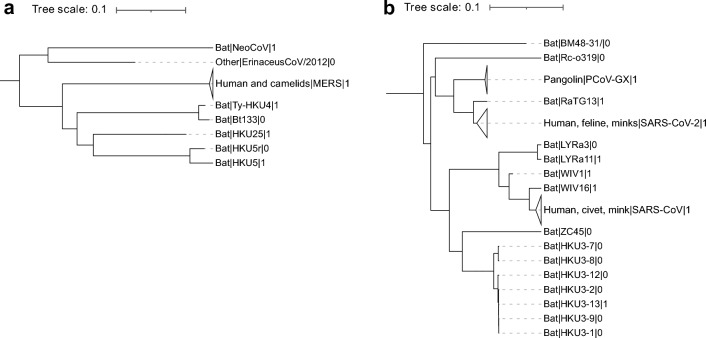
Phylogenetic tree for viruses related to Bt133, HKU5r and LYRa3 at the S gene. Pruned version of maximum-clade-credibility tree generated from 424 alpha and beta coronaviruses (full tree available in Supplementary Fig. S2). Each leaf shows the host, the name of the virus and the binding status separated by a pipe symbol. Non-human viruses with published binding annotation to a human receptor and human viruses have a binding status of 1 (0 otherwise). Solid gray triangles at the left of a leaf represent multiple variants in the particular leaf. (**a**) Phylogenetic tree for the Merbecovirus subgenus. Bat coronavirus Bt133 and HKU5r are phylogenetically related to Ty-HKU4 and HKU5r respectively. (**b**) Phylogenetic tree for the Sarbecovirus subgenus. LYRa3 is phylogenetically related to LYRa11.

Our method assigned a h-BiP score > 0.5 to Bt133, HKU5r and LYRa3, suggesting that these three viruses with unknown human binding status may bind to a human receptor. Bt133 is a beta coronavirus from the Merbecovirus subgenus, and it is phylogenetically related to Ty-HKU4 (see Fig. 3a), a bat coronavirus for which there is experimental evidence of binding to human receptor dipeptidyl peptidase 4 (hDPP4)^24, 36^. HKU5r also belongs to the Merbecovirus subgenus and it is phylogenetically related to HKU5 (Fig. 3a). While experimental evidence shows that HKU5 binds human cells, it is known that it does not bind hDPP4^24^. In fact, the specific human receptor of HKU5 still remains unknown^40^. LYRa3 is a beta coronavirus from the Sarbecovirus subgenus, and it is phylogenetically related to *Rhinolophus affinis* coronavirus isolate LYRa11^27^ (see Fig. 3b), which binds human receptor angiotensin-converting enzyme 2 (hACE2)^29, 31^. Host recognition and cell entry is mediated by the S protein^22–24^. The high sequence identity (97%) that the S protein of Bt133 shares with that of Ty-HKU4 suggests that Bt133 binds to hDPP4. Similarly, a 99% S protein sequence identity between LYRa3 and LYRa11 suggests that LYRa3 binds to hACE2. The S protein of HKU5r has a 93% sequence identity with that of HKU5. This high % sequence identity between HKU5 and HKU5r together with the high h-BiP value, suggests that HKU5r binds a human receptor different from hDDP4.

**Figure 4:**
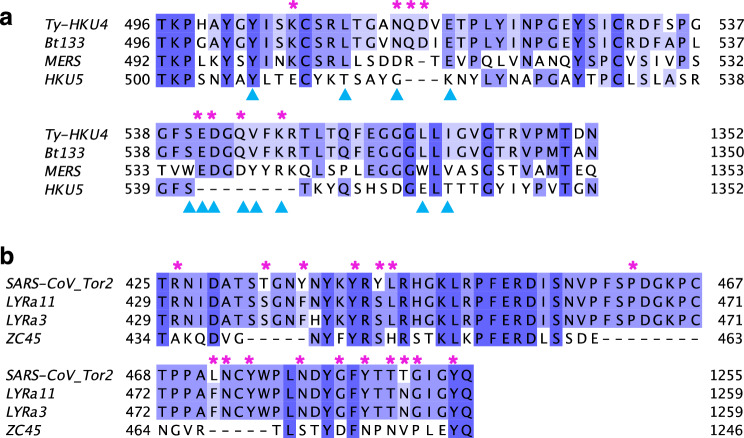
Multiple sequence alignment for phylogenetically related viruses at the RBM. Multiple sequence alignment was performed with MUSCLE^68^ and produce visualizations with Jalview^69^. A darker shade shows residues conserved in at least 50% of the sequences. (**a**) Comparison of viruses related to Bt133 within the Merbecovirus subgenus. Ty-HKU4 and MERS are viruses known to bind human receptor hDPP4. Experimental studies found no evidence of binding from HKU5 to hDPP4. Bt133 conserves all contact residues used by Ty-HKU4 to bind hDPP4 in 24 (marked with a pink asterisk). MERS uses four of the same contact residues^36^ than Ty-HKU4 (indicated by a blue triangle). HKU5, the only virus in the list unable to bind hDPP4, does not share any of the 8 contact residues from Ty-HKU4, and it shows several deletions at the RBM. (**b**) Comparison of viruses related to LYRa3 within the Sarbecovirus subgenus. LYRa11 is phylogenetically related to SARS-CoV and there is experimental evidence of binding from both to human receptor hACE2. LYRa11 conserves 12 (out of 17) of the contact residues used by SARS-CoV^29, 31^ (marked with a pink asterisk). At the RBM, LYRa3 differs from LYRa11 only at H441, which is not a contact residue used by SARS-CoV. Experimental studies found no evidence of binding from ZC45 to hACE2. ZC45 conserves only 2 out of the 17 contact residues from SARS-CoV, and it shows several deletions at the RBM.

The S protein binds to the human receptor through the receptor binding domain (RBD). The RBD is composed of a core domain and the receptor binding motif (RBM) that comes in direct contact with the host receptor^24^. A multiple sequence alignment of Bt133 with typical members of the Merbecovirus subgenus at the RBM (see Fig. 4a) revealed that Bt133 conserves all 8 contact residues used by Ty-HKU4 to bind hDPP4^24, 36^. Thus, suggesting that Bt133 binds hDPP4. Figure 4a also shows a region of the HKU5 genome that aligns with the RBM of Ty-HKU4. When compared to Ty-HKU4, this region of HKU5 shows two deletions and different amino acids at the contact residues. These observations are consistent with the lack of binding of HKU5 to hDPP4.

LYRa3 and LYRa11 are phylogenetically related to SARS-CoV Tor2, and both share 89.7% sequence identity at the S gene. A multiple sequence alignment for related viruses within the Sarbecovirus subgenus shows that LYRa11 and LYRa3 are identical at the RBM except at residue H441. Twelve of the 17 contact residues that SARS-CoV Tor2 uses to bind to hACE2 are conserved (see Fig. 4b). In contrast, experimental studies from bat coronavirus ZC45^45^ did not find evidence of binding to hACE2^29, 31^. When compared to SAR-CoV Tor2, ZC45 shows three deletions at the RBM and different amino acids at all locations of the contact residues of SAR-CoV Tor2, except for one at Y449. These results suggest that LYRa3 binds to hACE2.

### Molecular dynamics confirm binding of S to human receptors for coronaviruses Bt133 and LYRa3

Phylogenetic analysis, alignment results and the h-BiP score suggest that bat coronaviruses Bt133 and LYRa3 potentially bind to human receptors and are, therefore, candidates for host expansion to humans. We then used molecular dynamics simulations (MD) to validate binding in silico and to determine their contact residues. Three-dimensional structures of the receptor binding domain (RBD) bound to their corresponding human receptor for Lyra3 and Bt133 were obtained from crystal structure data and molecular modeling (see “Methods”).

The S protein of LYRa3 shares 99% identity with LYRa11 with one single point mutation at the RBD. Since LYRa11 is known to bind hACE2^29, 31^, we expect LYRa3 to have similar binding properties. Results from three independent simulations showed that the average H-bond count between the RBD of LYRa3 and hACE2 was 5.1 (see Table 4). Similarly, the average number of H-bonds between the RBD of LYRa11 and hACE2 was 5.8 H-bonds. The difference in the number of H-bonds between LYRa3 and LYRa11 was not statistically significant (p-value: 0.2947), suggesting that they have comparable binding energies. Two different estimations for the binding free energy (see “Methods”) of both complexes confirmed that the difference was not statistically significant (HawkDock p-value: 0.7599). A breakdown of the different types of interactions is available at Supplementary Table S1 (PRODIGY^46^) and Supplementary Table S2 (HawkDock^47^). The contact residues for LYRa3 were unknown. Therefore, we identified all of its contact residues during the course of the simulation (Supplementary Table S3). Our simulations revealed that contact residues G492, N477, T490, G486 and Y485 were present in at least 45% of the sampled conformations. A comprehensive list of all the bonds and their respective frequencies are available in (Supplementary Table S3).

**Table 4:** Average H-bonds between RBD and human receptor.

Virus	Sim1	Sim2	Sim3	Average (n = 3)	Std. error	p-value
LYRa11	6.6	5.8	5.1	5.8	0.75	
LYRa3	4.3	5.5	5.5	5.1	0.73	0.2947
Ty-HKU4	6.4	5.6	6.1	6.0	0.41	
Bt133	6.6	4.8	4.8	5.4	1.03	0.3780

Average number of H-bonds between the RBD and the human receptor by MD simulation (Sim1, Sim2, Sim3). The average was computed from all available sampled conformations after reaching equilibrium (4 ns). The average number of H-bonds of the three simulations and corresponding standard error is also shown. A t-test was performed to compare the means between LYRa11 and LYRa3 and between Ty-HKU4 and Bt133. The p-value corresponds to the two tailed test.

Next, we analyzed the interaction between the S protein of Bt133 and hDPP4. Despite containing 13 mutations in its RBD, alignment at the RBM showed that the contact residues of Ty-HKU4 are also present in Bt133. Results from three independent simulations showed that the average count of H-bonds was 5.4 for Bt133 and 6.0 for Ty-HKU4 (Table 4). The difference in average values was not statistically significant (p-value: 0.3780), suggesting that Bt133 and Ty-HKU4 have comparable binding energies when bound to hDPP4. The binding free energy from the two estimations of both complexes (Supplementary Tables S1 and S2) confirmed that the difference was not statistically significant (HawkDock p-value: 0.6009).

**Figure 5:**
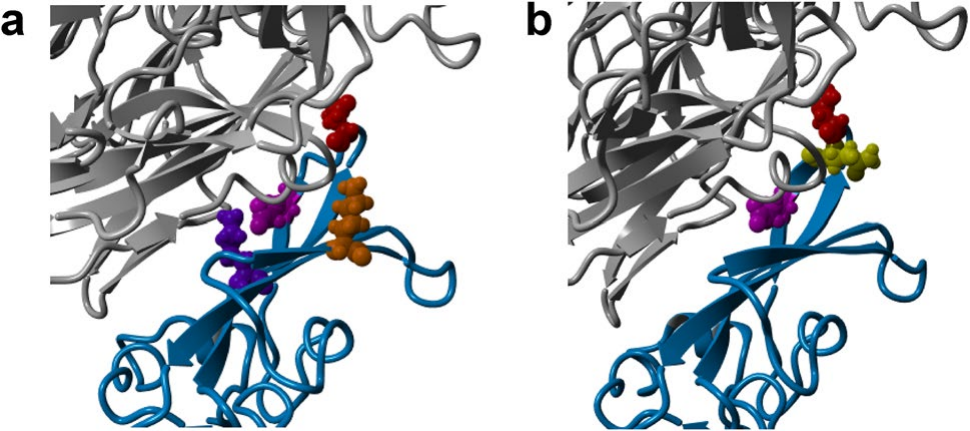
Most frequent contact residues for Ty-HKU4 and Bt133. The RBD is shown in light blue and the DPP4 human receptor in grey. Residues involved in frequent (average ≥  45% in Supplementary Tables S4 and S5) H-bonds are depicted in different colors. (**a**) Frequent contact residues for Ty-HKU4 are E518 (magenta), N514 (red), K506 (purple) and K547 (orange). (**b**) Frequent contact residues for Bt133 are E518 (magenta), N514 (red) and Q515 (yellow).

Our simulations confirmed all reported contact residues between Ty-HKU4 and hDPP4^24^ except for Q544. Our simulations also revealed contact residues N468, S465 and Y460, which have not been previously reported (Supplementary Table S4). Figure 5 shows contact residues for both Ty-HKU4 (a) and Bt133 (b). Only two contact residues were frequently observed in both viruses. A bond between E518 (magenta) in the RBD and Q344 in hDPP4, and between N514 (red) in the RBD and R317 in hDPP4, were present in at least 94% of the sampled conformations. Two additional contact residues were present in at least 50% of the sampled conformations from Ty-HKU4: K506 (purple) and K547 (orange). However, these two contact residues were present in less than 39% sampled conformations of Bt133 (Supplementary Table S5). Instead, only one additional contact residue was present for Bt133 in more than 70% of the sampled conformations: Q515 (yellow).

### The h-BiP score predicts binding of viruses that do not use the canonical receptor

Some coronaviruses are known not to bind canonical receptors^24, 39^. For instance, two recently reported MERS-related coronaviruses PDF2180 and NeoCoV have been shown to bind human ACE2 instead of hDPP4^39^. The h-BiP scores for these two betacoronaviruses were 0.95 and 0.88 respectively. These results combined with an h-BiP score of 0.98 for HKU5 (positive for binding) indicates that h-BiP can properly classify binding of coronaviruses to human receptor independently of the receptor preference of the virus.

### The h-BiP score predicts binding of SARS-CoV2 to hACE2

We explored whether the proposed method can be used to detect potential human-infection of novel coronaviruses. To test this hypothesis, we repeated the study and computed a h-BiP score for SARS-CoV-2 after excluding all SARS-CoV-2 viruses and all viral sequences uploaded to the database after Dec. 31, 2019 from the training set. This new data set consisted of 1272 viruses with 540 labeled as positive for binding to a human receptor. In this case the h-BiP value of SARS-CoV2 was 0.62. The model had sensitivity and specificity values of 99%. We conclude the proposed method would have predicted binding of SARS-CoV-2 to a human receptor in the early stages of the pandemic.

## Discussion

The COVID-19 pandemic has demonstrated the need to develop tools to predict spillover events. At the cellular and molecular level, three key steps, which are yet to be fully understood, are required for a spillover event: the evasion of the host’s immune system by the virus^4^, the infection of the cell by the virus^23, 24^ and the replication of the virus in the new host cell^4, 23^. In coronaviruses, the infection event is mediated by the binding of the viral spike (S) protein to the host receptor^23, 24^. The vast abundance of recently collected S protein data opens the way for machine learning (ML) methods that will help accelerate the pace of discovery of virus candidates for spillover events.

Here, we propose a new machine learning approach to predict the binding of alpha and beta coronaviruses to a human receptor. We call this model the human-Binding Potential (h-BiP). As an alignment-free approach, h-BiP overcomes limitations associated with multiple sequence alignment (msa) methods, such as the lack of robustness against genomic rearrangements or low computational efficiency (O(nm) for msa)^19^. While most alignment-free approaches rely on k-mer counts, which disregards their relative position in the genome^14^, h-BiP uses a neural network to create k-mer embeddings through a context window. These embeddings encode information about their neighboring k-mers in the sequence, and the resultant classifier is highly accurate.

Viruses with known human receptor are overrepresented in the group of viruses labeled as having evidence of binding and consequently the method has an inherent bias to correctly classify those viruses with known human receptor. To account for this bias, we included viruses with unknown human receptor. Our method benefits from this information which is in contrast with msa and biophysical methods that require knowledge on the position of the RBM or binding residues.

The h-BiP score is highly correlated with % identity against human coronaviruses. Yet, the classifier discriminates among viruses with similar % identity and identifies viruses with low % identity that may bind to human receptors. Such is the case of bat coronavirus LYRa3, which has a similar % identity value to other viruses with unknown binding status to human receptor such as HKU14; and the cases of Bt133 and Pipistrellus abramus HKU5-related virus (HKU5r), with low % identity (67.2% and 64.1% resp.). However, the three of them have an h-BiP score greater than 0.5. The method suggests that these viruses with previously unknown binding status may bind to a human receptor. Phylogenetic analysis revealed that they are closely related to viruses known to bind human receptors. A pairwise alignment between the RBDs of Ty-HKU4 and Bt133 revealed that Bt133 conserves all 8 contact residues used by Ty-HKU4 to bind hDPP4 despite having 13 mutations in the RBD. On the other hand, with the exception of residue 441, the protein sequences of LYRa3 and LYRa11 are identical at the RBM. These findings suggest that Bt133 and LYRa11 bind to hDPP4 and hACE2, respectively.

We detected HKU5r, with a h-BiP score of 0.99. HKU5r has a 93% identity in the S protein with HKU5. Interestingly, HKU5 is a Merbecovirus for which the human receptor is unknown and it has been shown not to bind to hDPP4^24^. This is one example on how the prediction of our method improves when we add viruses for which the only information known is whether they bind or not to any human receptor. The absence of a known human receptor for HKU5 limits the possibility of further biophysical studies of binding hence we did not pursue them here.

We confirmed binding of Bt133 and LYRa3 in silico through a combination of structural modeling and molecular dynamics simulations. The average number of contact residues from the simulations of both viruses was not statistically different from that of Ty-HKU4 and LYRa11, respectively. We used HawkDock and PRODIGY to estimate binding energies. We selected HawkDock’s molecular mechanics/generalized Born surface area (MM/GBSA) for the calculations. MM/GBSA overestimated the magnitude of binding free energy but the obtained values correlate well with H-bond counts^48^. While PRODIGY results provided closer estimates for binding energies measured in in-vitro studies^48^, it did not discriminate well among variants. The binding energy estimation from Bt133 and LYRa3 was not statistically different from that of Ty-HKU4 and LYRa11, respectively. These results suggest they have comparable binding energies. We identified all contact residues of Bt133 and Ty-HKU4 (Supplementary Tables S4 and S5). Only contact residues E518 and N514 were frequently used (> 94% of sampled conformations) by both viruses to bind to the human receptor. Contact residue E518 is also known to be relevant for binding of MERS^24, 36^ to hDPP4. Our simulations also revealed three previously unreported contact residues from Ty-HKU4 (N468, S465 and Y460). Our study also identified the contact residues of LYRa3 (Supplementary Table S3).

As expected with any classifier, h-BiP produced a few false negatives in our dataset. Such is the case of HKU3-13 (h-BiP = 0.2), which is the only known member from the HKU3 clade experimentally confirmed for human binding in our dataset. Interestingly, HKU3-8 has been reported not to bind human receptors^38^.

We also tested the proposed method under the scenario for the emergence of a novel virus. In particular, we asked whether h-BiP would predict the binding of a novel coronavirus such as SARS-CoV-2. In the absence of all SARS-CoV-2 viruses and any viral sequence uploaded after its publication, the h-BiP score for the wild type of SARS-CoV-2 was 0.62, demonstrating that h-BiP may be a valuable tool to detect the potential of a virus to cross species and originate an epidemic.

## Methods

### Datasets

On 11/05/2020, we downloaded 28,368 RNA spike protein sequences of all alpha and beta coronaviruses from the NCBI Virus^49^ database. Compared to the number of full nucleotide sequences, the number of annotated sequences for the Sarbecovirus genus (excluding SARS-CoV-2) was limited. On 07/26/2021, we downloaded all available nucleotide sequences and extracted the S protein (see section “Extracting the S protein from full sequences” for details), expanding the data from 78 to 194 unique sequences from the Sarbecovirus genus (excluding SARS-CoV-2). To reduce the impact of an unbalanced dataset, we randomly removed 50% of SARS-CoV-2 (under-sampling), preserving reference sequences. The final alpha and beta coronavirus dataset consisted of 2,534 amino acid sequences. We curated the host field by combining information from isolation source, submission notes and the related publication. We also removed 7 viruses that were genetically modified (Accessions: FJ882951, FJ882957, HQ890538, FJ882942, HQ890534, MT782114, MT782115) as well as a draft virus from a pangolin (MT084071). Sequences in the data set were annotated as positive or negative for human binding. Human coronaviruses, and viruses from non-human animal hosts with published binding evidence to a human receptor, were labeled as positive for binding (n = 1735). Viruses from non-human hosts reported as unable to bind human receptors, and those for which the binding condition was unknown, were labeled as negative for binding (n = 799). A comprehensive list of viruses with confirmed binding status is available in Table 1. The final dataset, according to binding status, is available in Table 5.

**Table 5:** Final dataset by binding condition to human receptor.

Host	Negative/unknown	Positive	Total	
Human	0	1530	1530	60.4%
Non-human	799	205	1004	39.7%
Total	31.5%	68.5%	2534	

### Extracting the S protein from full sequences

On 07/26/2021, we downloaded all available nucleotide (nt) sequences from the Sarbecovirus genus (excluding SARS-CoV-2) for a total of 643 sequences with at least 1603 nt of length. The S protein contains two subunits: the S1 subunit containing the receptor-binding-domain, and the S2 subunit, which mediates membrane fusion^50, 51^. Prior to SARS-CoV-2 emergence, a highly conserved domain across all coronaviruses (SFIEDLLFNKVTLADAGF, NCBI accession cl40439) was found in the S2 subunit^51^. In order to extract the S protein from the full RNA sequence, we first translated it into the three possible reading frames. Next, we located the conserved domain by searching for the “SFIEDLLFN” motif (allowing for at most one substitution). The final putative S protein was the sequence enclosed by the start and stop codons, which contained the “SFIEDLLFN” motif. A total of 554 S proteins (194 unique) were found and included in the final dataset. We tested different motif lengths and number of substitutions to locate the conserved domain, but 9 amino acids, and 1 substitution, were found to be optimal.

### Training and test sets

The final dataset of 2,534 sequences was split into a training (85%) and test set (15%), stratified by the following groups: hCoV-OC43, hCoV-HKU1, MERS, SARS-CoV-1, SARS-CoV-2, hCoV-NL63, hCoV-229E, other MERS-related, other Sarbecovirus, other Betacoronavirus, porcine epidemic diarrhea virus, and other Alphacoronavirus.

### Sequence embedding

In order to generate n-dimensional vectors from the protein sequences, we first built “words” of 3 letters (trimers) with 3 contiguous amino acids. We considered all possible trimers by sliding a window one amino acid at a time as shown in Fig. 1. To produce trimer embeddings, we used a skip-gram with negative sampling (vector-size = 100, context-size = 25, negative samples = 1) as in 52. Finally, each sequence was represented as the sum of vectors from all of its trimers. That is, if a particular sequence (*seq*_*k*_) consisted of *n* trimers {*w*_*1*_*, w*_*2*_*, …, w*_*n*_}, and each trimer was represented by a 100-dimensional vector *w*_*i*_ = [*x*_*i_1*_*, x*_*i_2*_*, …, x*_*i_100*_], then the final sequence embedding was calculated from Eq. (1).

1 $${seq}_{k}={\sum }_{i=1}^{n}{w}_{i}$$

### Classification

In order to classify the sequences according to their potential to bind a human receptor, we labeled them as positive or negative for human binding as described in “Datasets” section. Normalized sequence embeddings of the training set were used to create a logistic regression model. The model used a ridge classifier with C = 1.5 inverse regularization strength and the limited memory BFGS (L-BFGS) optimization algorithm. A virus was classified as positive for binding if the score was 0.5 or higher. The final classifier was invariant to the threshold value (see Supplementary Fig. S1).

### Phylogenetic tree

A phylogenetic tree for the S protein of alpha and beta coronaviruses was generated from a subset of 424 sequences (out of 2534) from the original dataset. This subset includes all reference viruses from both genera, several members from each of the 7 human coronaviruses and all viruses from non-human hosts with positive or negative published human binding annotation (see Table 1). A comprehensive list is available in Supplementary Table S4. A multiple sequence alignment (msa) of all sequences in the subset was generated with BBMap^53^. We used BEAST^54^ on the msa to reconstruct multiple phylogenetic trees with a log-normal distributed relaxed molecular clock and a non-parametric coalescent prior. The final tree shown in Supplementary Fig. S2 corresponds to the maximum-clade-credibility tree. We used iTOL^55^ for tree visualization and annotation.

### Candidate structures for molecular dynamics

The starting RBD structures for molecular dynamics (MD) simulations were obtained using PDB file 4QZV^24^ of Ty-HKU4- hDPP4 complex. At the RBD, Bt133 differs from Ty-HKU4 in 13 residues. These mutations were added to PDB 4QZV^24^ using YASARA^56^. The starting RBD structures for the RBD of LYRa3 and LYRa11 were generated using AlphaFold^57^ (implemented within the ColabFold suite^58^). Due to the absence of experimentally determined LYRa3-hACE2 and LYRa11-hACE2 complexes, we used the SARS-CoV-hACE2 complex (PDB 2AJF^59^) as a template in AlphaFold. The resulting structures were aligned to SARS-CoV using the MUSTANG^60^ method in YASARA^56^. These alignments were in strong agreement (< 2.0A) and used as starting structures in our simulations.

### Molecular dynamics

To simulate protein–protein interactions, we used the molecular-modelling package YASARA^56^ to substitute individual residues and to search for minimum-energy conformations on the resulting modified candidate structures. For all structures, we carried out an energy minimization (EM) routine, which included steepest descent and simulated annealing minimization (until free energy stabilizes to within 50 J/mol) to remove clashes. All MD simulations were run using the AMBER14 force field^61^ for solute, GAFF2^62^ and AM1BCC^63^ for ligands and TIP3P^63^ for water. The cutoff was 8 Å for Van der Waals forces (AMBER’s default value^64^), and no cutoff was applied for electrostatic forces (using the Particle Mesh Ewald algorithm^65^). The equations of motion were integrated with a multiple timestep of 1.25 fs for bonded interactions and 2.5 fs for non-bonded interactions at T = 298 K and P = 1 atm (NPT ensemble) via algorithms described in 67. Prior to counting the RBD’s hydrogen bonds and calculating the free energy, we carried out several pre-processing steps on the structure, including an optimization of the hydrogen-bonding network^66^ to increase the solute stability and a pKa prediction to fine-tune the protonation states of protein residues at the chosen pH of 7.4^67^. Insertions and mutations were carried out using YASARA’s BuildLoop and SwapRes commands^67^, respectively.

Structure conformations from the simulations were collected every 100 ps after 4 ns of equilibration time as determined by the solute root mean square deviations (RMSDs) from the starting structure. For all bound structures, we ran the simulations for at least 10 ns post equilibrium and verified stability of time series for RBD-receptor hydrogen bond counts and root mean square deviation (RMSD) from these starting structures. Hydrogen bonds (H-bonds) were counted and tabulated using a distance and an angle approximation between donor and acceptor atoms as described in 66. It is important to note in this approach, salt bridges of proximate residues are effectively counted as H-bonds between basic side chain amide groups and acidic side chain carboxyl groups. Therefore, ionic interactions are also included in the H-bond count. In a previous publication, we show that the H-bond count, as defined here, correlates well with binding free energy estimates that were obtained using the molecular mechanics/generalized Born surface area method.

### Average number of H-bonds

In previous studies, we have shown that the average number of H-Bonds correlates well with binding energy^48^. Therefore, for each MD simulation, we recorded the number of H-bonds formed between protein–protein interactions (including ionic bonds) at each sampled conformation (snapshots). The average number of H-bonds was computed from all available sampled conformations after reaching equilibrium (4 ns). We performed three independent MD simulations from each complex and determined the grand average and standard error. Results are available in Table 4.

### H-bond frequencies (%)

At each sampled conformation (snapshot) from a MD simulation, we tracked every H-bond (including ionic bonds) and recorded the participant amino acids from the ligand and receptor. We computed the frequency for each pair dividing the number of sampled conformations where the pair was present by the total number of sampled conformations (double bonds are not considered in this count) and multiplied by 100. H-bond frequencies (%) from every simulation are available in the Supplementary Information S1.

### Prediction of binding affinity

To estimate the binding free energy for each of the RBD-receptor complexes we used the energy-minimized structures on HawkDock^47^and PRODIGY^46^ servers. We selected the molecular mechanics/generalized Born surface area (MM/GBSA) method in HawkDock. For every simulation on RBD-receptor pairs we average over 3 snapshots of equilibrium conformations.

### Human and animal subjects

No human or animal subjects were directly involved in this study.

## Supplementary Information

Supplementary Information.

## Data Availability

The datasets and corresponding GenBank accessions used to create h-BiP, together with the resultant scores, are available at https://github.com/Arsuaga-Vazquez-Lab/h-BiP.

## Code availability

The h-BiP package and Python source code are available at https://github.com/Arsuaga-Vazquez-Lab/h-BiP.

## References

[1] Cui J, Li F, Shi ZL (2019). Origin and evolution of pathogenic coronaviruses. Nat. Rev. Microbiol..

[2] Naguib MM, Ellström P, Järhult JD, Lundkvist Å, Olsen B (2020). Towards pandemic preparedness beyond COVID-19. The Lancet Microbe.

[3] Olival KJ, Hosseini PR, Zambrana-Torrelio C, Ross N, Bogich TL, Daszak P (2017). Host and viral traits predict zoonotic spillover from mammals. Nature.

[4] Plowright RK (2017). Pathways to zoonotic spillover. Nat. Rev. Microbiol..

[5] Rodriguez-Morales AJ (2020). History is repeating itself: Probable zoonotic spillover as the cause of the 2019 novel Coronavirus Epidemic. Infez. Med..

[6] Gorbalenya, A.E. *et al.* Severe acute respiratory syndrome-related coronavirus: The species and its viruses—a statement of the Coronavirus Study Group. *BioRxiv* (2020).

[7] Fehr, A.R., & Perlman, S. Coronaviruses: an overview of their replication and pathogenesis. *Coronaviruses* 1–23 (2015). 10.1007/978-1-4939-2438-7_1 PMC4369385 25720466

[8] Hu B (2017). Discovery of a rich gene pool of bat SARS-related coronaviruses provides new insights into the origin of SARS coronavirus. PLoS Pathog..

[9] Lamy-Besnier Q, Brancotte B, Ménager H, Debarbieux L (2021). Viral Host Range database, an online tool for recording, analyzing and disseminating virus–host interactions. Bioinformatics.

[10] Wang, W. *et al.* A network-based integrated framework for predicting virus–prokaryote interactions. *NAR Genom. Bioinf.* **2**(2), p.lqaa044 (2020). 10.1093/nargab/lqaa044 PMC7324143 32626849

[11] Grange, Z.L. *et al.* Ranking the risk of animal-to-human spillover for newly discovered viruses*. Proc. Natl. Acad. Sci.* **118**(15) (2021). 10.1073/pnas.2002324118 PMC8053939 33822740

[12] Sánchez, C.A., Li, H., Phelps, K.L., Zambrana-Torrelio, C., Wang, L.F., Olival, K.J., & Daszak, P. A strategy to assess spillover risk of bat SARS-related coronaviruses in Southeast Asia. *MedRxiv* (2021). 10.1038/s41467-022-31860-w PMC9363439 35945197

[13] Xu B, Tan Z, Li K, Jiang T, Peng Y (2017). Predicting the host of influenza viruses based on the word vector. PeerJ.

[14] Mock F, Viehweger A, Barth E, Marz M (2021). VIDHOP, viral host prediction with Deep Learning. Bioinformatics.

[15] Zhang M, Yang L, Ren J, Ahlgren NA, Fuhrman JA, Sun F (2017). Prediction of virus-host infectious association by supervised learning methods. BMC Bioinf..

[16] Gałan W, Bąk M, Jakubowska M (2019). Host taxon predictor-a tool for predicting taxon of the host of a newly discovered virus. Sci. Rep..

[17] Bartoszewicz, J.M., Seidel, A., & Renard, B.Y. Interpretable detection of novel human viruses from genome sequencing data. *NAR Genom. Bioinf.* **3(1),** lqab004 (2021). 10.1093/nargab/lqab004 PMC7849996 33554119

[18] Li H, Sun F (2018). Comparative studies of alignment, alignment-free and SVM based approaches for predicting the hosts of viruses based on viral sequences. Sci. Rep..

[19] Zielezinski A, Vinga S, Almeida J, Karlowski WM (2017). Alignment-free sequence comparison: benefits, applications, and tools. Genome Biol..

[20] Chowdhury B, Garai G (2017). A review on multiple sequence alignment from the perspective of genetic algorithm. Genomics.

[21] Chan JM, Carlsson G, Rabadan R (2013). Topology of viral evolution. Proc. Natl. Acad. Sci..

[22] Liu K, Pan X, Li L, Yu F, Zheng A, Du P, Han P, Meng Y, Zhang Y, Wu L, Chen Q (2021). Binding and molecular basis of the bat coronavirus RaTG13 virus to ACE2 in humans and other species. Cell.

[23] Li Y, Wang H, Tang X, Fang S, Ma D, Du C, Wang Y, Pan H, Yao W, Zhang R, Zou X (2020). SARS-CoV-2 and three related coronaviruses utilize multiple ACE2 orthologs and are potently blocked by an improved ACE2-Ig. J. Virol..

[24] Wang Q, Qi J, Yuan Y, Xuan Y, Han P, Wan Y, Ji W, Li Y, Wu Y, Wang J, Iwamoto A (2014). Bat origins of MERS-CoV supported by bat coronavirus HKU4 usage of human receptor CD26. Cell Host Microbe.

[25] Tang XC (2006). Prevalence and genetic diversity of coronaviruses in bats from China. J. Virol..

[26] Li B (2020). Discovery of bat coronaviruses through surveillance and probe capture-based next-generation sequencing. Msphere.

[27] He B (2014). Identification of diverse alphacoronaviruses and genomic characterization of a novel severe acute respiratory syndrome-like coronavirus from bats in China. J. Virol..

[28] Mikolov, T., Chen, K., Corrado, G., & Dean, J. Efficient estimation of word representations in vector space. *arXiv preprint* arXiv:1301.3781 (2013).

[29] Murakami S (2020). Detection and characterization of bat sarbecovirus phylogenetically related to SARS-CoV-2 Japan. Emerg. Infect. Dis..

[30] Zhang S (2021). Bat and pangolin coronavirus spike glycoprotein structures provide insights into SARS-CoV-2 evolution. Nat. Commun..

[31] Letko M, Marzi A, Munster V (2020). Functional assessment of cell entry and receptor usage for SARS-CoV-2 and other lineage B betacoronaviruses. Nat. Microbiol..

[32] Zheng M (2020). Bat SARS-Like WIV1 coronavirus uses the ACE2 of multiple animal species as receptor and evades IFITM3 restriction via TMPRSS2 activation of membrane fusion. Emerg. Microbes Infect..

[33] Dixson JD, Azad RK (2021). A novel predictor of ACE2-binding ability among betacoronaviruses. Evol. Med. Public Health.

[34] Wu F (2020). A new coronavirus associated with human respiratory disease in China. Nature.

[35] Seifert, S.N., & Letko, M.C. A sarbecovirus found in Russian bats uses human ACE2. *bioRxiv* (2021).

[36] Lau SK, Zhang L, Luk HK, Xiong L, Peng X, Li KS, He X, Zhao PSH, Fan RY, Wong AC, Ahmed SS (2018). Receptor usage of a novel bat lineage C betacoronavirus reveals evolution of Middle East respiratory syndrome-related coronavirus spike proteins for human dipeptidyl peptidase 4 binding. J. Infect. Dis..

[37] Cheng Y, He B, Yang J, Ye F, Lin S, Yang F, Chen Z, Chen Z, Cao Y, Lu G (2019). Crystal structure of the S1 subunit N-terminal domain from DcCoV UAE-HKU23 spike protein. Virology.

[38] Khaledian E (2022). Sequence determinants of human-cell entry identified in ACE2-independent bat sarbecoviruses: A combined laboratory and computational network science approach. EBioMedicine.

[39] Xiong, Q. *et al.* Close relatives of MERS-CoV in bats use ACE2 as their functional receptors. *Nature* 1–10 (2022). 10.1038/s41586-022-05513-3 PMC9734910 36477529

[40] Guo H (2022). ACE2-independent bat sarbecovirus entry and replication in human and bat cells. MBio.

[41] Chu (2018). MERS coronaviruses from camels in Africa exhibit region-dependent genetic diversity. Proc. Natl. Acad. Sci..

[42] Sabir JS (2016). Co-circulation of three camel coronavirus species and recombination of MERS-CoVs in Saudi Arabia. Science.

[43] Altschul SF, Madden TL, Schäffer AA, Zhang J, Zhang Z, Miller W, Lipman DJ (1997). Gapped BLAST and PSI-BLAST: A new generation of protein database search programs. Nucleic Acids Res..

[44] Jin L (2007). Analysis of the genome sequence of an alpaca coronavirus. Virology.

[45] Hu D (2018). Genomic characterization and infectivity of a novel SARS-like coronavirus in Chinese bats. Emerg. Microbes Infect..

[46] Honorato RV (2021). Structural biology in the clouds: The WeNMR-EOSC ecosystem. Front. Mol. Biosci..

[47] Weng G (2019). HawkDock: A web server to predict and analyze the protein–protein complex based on computational docking and MM/GBSA. Nucleic Acids Res..

[48] Jawaid, M.Z. *et al.* Computational study of the furin cleavage domain of SARS-CoV-2: Delta binds strongest of extant variants. *bioRxiv* (2022).

[49] Brister JR, Ako-Adjei D, Bao Y, Blinkova O (2015). NCBI viral genomes resource. Nucleic Acids Res..

[50] Huang Y, Yang C, Xu XF, Xu W, Liu SW (2020). Structural and functional properties of SARS-CoV-2 spike protein: Potential antivirus drug development for COVID-19. Acta Pharmacol. Sin..

[51] Madu IG, Roth SL, Belouzard S, Whittaker GR (2009). Characterization of a highly conserved domain within the severe acute respiratory syndrome coronavirus spike protein S2 domain with characteristics of a viral fusion peptide. J. Virol..

[52] Asgari E, Mofrad MR (2015). Continuous distributed representation of biological sequences for deep proteomics and genomics. PLoS ONE.

[53] Bushnell, B. BBMap: a fast, accurate, splice-aware aligner (No. LBNL-7065E*). Lawrence Berkeley National Lab. (LBNL)*, Berkeley, CA (United States) (2014).

[54] Bouckaert, R. *et al.* BEAST 2.5: An advanced software platform for Bayesian evolutionary analysis. *PLoS Comput. Biol.* **15**(4), e1006650 (2019). 10.1371/journal.pcbi.1006650 PMC6472827 30958812

[55] Letunic I, Bork P (2007). Interactive Tree Of Life (iTOL): An online tool for phylogenetic tree display and annotation. Bioinformatics.

[56] Krieger E, Vriend G (2014). YASARA view—molecular graphics for all devices—from smartphones to workstations. Bioinformatics.

[57] Evans, R. *et al.* Protein complex prediction with AlphaFold-Multimer. BioRxiv (2021).

[58] Mirdita, M., Schütze, K., Moriwaki, Y., Heo, L., Ovchinnikov, S., & Steinegger, M. ColabFold-Making protein folding accessible to all (2021). 10.1038/s41592-022-01488-1 PMC9184281 35637307

[59] Li F, Li W, Farzan M, Harrison SC (2005). Structure of SARS coronavirus spike receptor-binding domain complexed with receptor. Science.

[60] Konagurthu AS, Whisstock JC, Stuckey PJ, Lesk AM (2006). MUSTANG: A multiple structural alignment algorithm. Proteins Struct. Funct. Bioinf..

[61] Maier JA, Martinez C, Kasavajhala K, Wickstrom L, Hauser KE, Simmerling C (2015). ff14SB: Improving the accuracy of protein side chain and backbone parameters from ff99SB. J. Chem. Theory Comput..

[62] Wang J, Wolf RM, Caldwell JW, Kollman PA, Case DA (2004). Development and testing of a general amber force field. J. Comput. Chem..

[63] Jakalian A, Jack DB, Bayly CI (2002). Fast, efficient generation of high-quality atomic charges. AM1-BCC model: II. Parameterization and validation. J. Comput. Chem..

[64] Hornak V, Abel R, Okur A, Strockbine B, Roitberg A, Simmerling C (2006). Comparison of multiple Amber force fields and development of improved protein backbone parameters. Proteins Struct. Funct. Bioinf..

[65] Essmann U, Perera L, Berkowitz ML, Darden T, Lee H, Pedersen LG (1995). A smooth particle mesh Ewald method. J. Chem. Phys..

[66] Krieger E, Darden T, Nabuurs SB, Finkelstein A, Vriend G (2004). Making optimal use of empirical energy functions: Force-field parameterization in crystal space. Proteins Struct. Funct. Bioinf..

[67] Krieger E, Vriend G (2015). New ways to boost molecular dynamics simulations. J. Comput. Chem..

[68] Edgar RC (2004). MUSCLE: A multiple sequence alignment method with reduced time and space complexity. BMC Bioinf..

[69] Waterhouse AM, Procter JB, Martin DM, Clamp M, Barton GJ (2009). Jalview Version 2—a multiple sequence alignment editor and analysis workbench. Bioinformatics.

