# Supplementary material for: Using machine learning to detect coronaviruses potentially infectious to humans
Source: Sci Rep. 2023 Jun 8;13:9319. doi: 10.1038/s41598-023-35861-7 (PMC10248971; doi:10.1038/s41598-023-35861-7)
Supplement: Supplementary file 1 — Supplementary Information. [file 41598_2023_35861_MOESM1_ESM.docx]

**Using machine learning to detect coronaviruses potentially infectious to humans**

Gonzalez-Isunza *et al.*

**Supplementary Information**


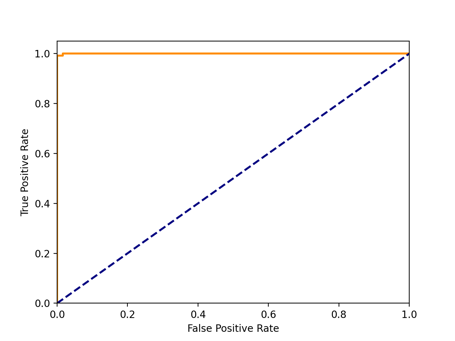


**Supplementary Fig. 1:** **Receiver Operating Characteristic curve (ROC) from h-BiP scores.** The ROC curve on the alpha and beta coronaviruses test set at different thresholds of the h-BiP scores is shown in orange. The expected behavior of a random classifier is depicted with a blue dashed line. The area under the curve has a value of 0.999 showing that the performance of the model is invariant to the threshold’s choice.

**a**

**b**

**Supplementary Fig. 2: Phylogenetic tree for alpha and beta coronaviruses at the S gene**.

Amino acid sequences from the S gene of 424 alpha and beta coronaviruses (Supplementary Table 6) were aligned using BBMap. The phylogenetic tree was generated using maximum-clade-credibility tree with BEAST and visualized with iTOL. Each leaf shows the accession number from NCBI, the host, the GenBank Title and the label for the binding status separated by a pipe symbol (1 if there is experimental evidence of binding to human receptor, 0 otherwise). Collapsed clades show the total number of members in parenthesis **a.** Alphacoronavirus genus **b.** Betacoronavirus genus

**Supplementary Table 1: Prediction of binding affinity of RBD-receptor complexes by PRODIGY**

| **HKU4-hDPP4** | **Sim1** | **Sim2** | **Sim3** | **Average** | **Std. error** |
| --- | --- | --- | --- | --- | --- |
| ΔG (kcal mol^-1^) | -10.1 | -10.1 | -10.4 | -10.2 | 0.17 |
| K_d_ (M) at ℃ | 3.70E-08 | 4.10E-08 | 2.30E-08 | 3.37E-08 | 9.45E-09 |
| ICs charged-charged | 4 | 4 | 3 | 3.67 | 0.58 |
| ICs charged-polar | 10 | 10 | 11 | 10.33 | 0.58 |
| ICs charged-apolar | 16 | 17 | 21 | 18.00 | 2.65 |
| ICs polar-polar | 5 | 5 | 6 | 5.33 | 0.58 |
| ICs polar-apolar | 14 | 13 | 14 | 13.67 | 0.58 |
| ICs apolar-apolar | 11 | 10 | 11 | 10.67 | 0.58 |
| NIS charged | 23.89 | 24.05 | 24.03 | 23.99 | 0.09 |
| NIS apolar | 35.83 | 35.36 | 35.34 | 35.51 | 0.28 |
|  |  |  |  |  |  |
| **Bt133-hDPP4** | **Sim1** | **Sim2** |  | **Average** | **Std. error** |
| ΔG (kcal mol^-1^) | -10.8 | -10.3 |  | -10.55 | 0.35 |
| K_d_ (M) at ℃ | 1.20E-08 | 2.90E-08 |  | 2.05E-08 | 1.20E-08 |
| ICs charged-charged | 4 | 4 |  | 4.00 | 0.00 |
| ICs charged-polar | 11 | 9 |  | 10.00 | 1.41 |
| ICs charged-apolar | 16 | 17 |  | 16.50 | 0.71 |
| ICs polar-polar | 6 | 5 |  | 5.50 | 0.71 |
| ICs polar-apolar | 18 | 15 |  | 16.50 | 2.12 |
| ICs apolar-apolar | 12 | 11 |  | 11.50 | 0.71 |
| NIS charged | 23.9 | 23.93 |  | 23.92 | 0.02 |
| NIS apolar | 35.91 | 36.73 |  | 36.32 | 0.58 |
|  |  |  |  |  |  |
| **LYRa11-hACE2** | **Sim1** | **Sim2** | **Sim3** | **Average** | **Std. error** |
| ΔG (kcal mol^-1^) | -11.1 | -9.6 | -10.3 | -10.33 | 0.75 |
| K_d_ (M) at ℃ | 7.20E-09 | 9.50E-08 | 2.90E-08 | 4.37E-08 | 4.57E-08 |
| ICs charged-charged | 1 | 1 | 2 | 1.33 | 0.58 |
| ICs charged-polar | 7 | 7 | 8 | 7.33 | 0.58 |
| ICs charged-apolar | 17 | 12 | 17 | 15.33 | 2.89 |
| ICs polar-polar | 2 | 2 | 2 | 2.00 | 0.00 |
| ICs polar-apolar | 17 | 13 | 14 | 14.67 | 2.08 |
| ICs apolar-apolar | 12 | 12 | 13 | 12.33 | 0.58 |
| NIS charged | 25.99 | 25.45 | 25.16 | 25.53 | 0.42 |
| NIS apolar | 34.87 | 35.89 | 36.7 | 35.82 | 0.92 |
|  |  |  |  |  |  |
| **LYRa3-hACE2** | **Sim1** | **Sim2** | **Sim3** | **Average** | **Std. error** |
| ΔG (kcal mol^-1^) | -9.5 | -10.3 | -10.1 | -9.97 | 0.42 |
| K_d_ (M) at ℃ | 1.00E-07 | 2.90E-08 | 3.70E-08 | 5.53E-08 | 3.89E-08 |
| ICs charged-charged | 2 | 1 | 3 | 2.00 | 1.00 |
| ICs charged-polar | 5 | 5 | 7 | 5.67 | 1.15 |
| ICs charged-apolar | 13 | 16 | 14 | 14.33 | 1.53 |
| ICs polar-polar | 2 | 2 | 2 | 2.00 | 0.00 |
| ICs polar-apolar | 12 | 15 | 14 | 13.67 | 1.53 |
| ICs apolar-apolar | 12 | 12 | 12 | 12.00 | 0.00 |
| NIS charged | 25.45 | 25.94 | 25.83 | 25.74 | 0.26 |
| NIS apolar | 35.89 | 36.29 | 35.93 | 36.04 | 0.22 |

Average results for each of the independent MD simulations (Sim1 to Sim 3) of the virus RBD bound to corresponding human receptor (details in Methods). ΔG: predicted binding affinity, K_d_: dissociation constant, ICs: number of Interfacial Contacts classified according to the nature of the interacting residues (polar/apolar/charged), NIS: non-interacting surfaces.

**Supplementary Table 2: Prediction of binding affinity of RBD-receptor complexes by HawkDock (MM/GBSA)**

| **HKU4-hDPP4** | **VDW** | **ELE** | **GB** | **SA** | **TOTAL**  **(kcal mol^-1^)** |
| --- | --- | --- | --- | --- | --- |
| Sim1 | -88.42 | -265.66 | 323.22 | -11.81 | -42.67 |
| Sim2 | -80.34 | -267.6 | 318.38 | -10.35 | -39.92 |
| Sim3 | -89.48 | -314.89 | 360.29 | -12.18 | -56.25 |
| **Average** | **-86.08** | **-282.72** | **333.96** | **-11.45** | **-46.28** |
| **Std. error** | **5.00** | **27.88** | **22.93** | **0.97** | **8.74** |
|  |  |  |  |  |  |
| **Bt133-hDPP4** | **VDW** | **ELE** | **GB** | **SA** | **TOTAL**  **(kcal mol^-1^)** |
| Sim1 | -87.09 | -170.45 | 217.13 | -11.78 | -52.19 |
| Sim2 | -82.92 | -110.30 | 163.67 | -10.85 | -40.40 |
| Sim3 | -79.71 | -116.69 | 173.05 | -10.31 | -33.65 |
| **Average** | **-83.24** | **-132.48** | **184.62** | **-10.98** | **-42.08** |
| **Std. error** | **3.70** | **33.04** | **28.55** | **0.74** | **9.38** |
|  |  |  |  |  |  |
| **LYRa11-hACE2** | **VDW** | **ELE** | **GB** | **SA** | **TOTAL**  **(kcal mol^-1^)** |
| Sim1 | -95.91 | -404.56 | 455.19 | -11.95 | -57.23 |
| Sim2 | -91.71 | -408.14 | 465.44 | -11.3 | -45.71 |
| Sim3 | -86.34 | -433.15 | 476.36 | -11.02 | -54.15 |
| **Average** | **-91.32** | **-415.28** | **465.66** | **-11.42** | **-52.36** |
| **Std. error** | **4.80** | **15.58** | **10.59** | **0.48** | **5.96** |
|  |  |  |  |  |  |
| **LYRa3-hACE2** | **VDW** | **ELE** | **GB** | **SA** | **TOTAL**  **(kcal mol^-1^)** |
| Sim1 | -85.91 | -432.18 | 479.49 | -10.19 | -48.78 |
| Sim2 | -90.13 | -368.53 | 419.37 | -10.71 | -50 |
| Sim3 | -87.78 | -443.26 | 487.73 | -11.21 | -54.52 |
| **Average** | **-87.94** | **-414.66** | **462.20** | **-10.70** | **-51.10** |
| **Std. error** | **2.11** | **40.33** | **37.32** | **0.51** | **3.02** |

Average results for each of the independent MD simulations (Sim1 to Sim 3) of the virus RBD bound to corresponding human receptor (details in Methods). Contributions to the free energy of binding: VDW: Van Der Waals, ELE: electrostatic, GB: Polar Solvation, SA: Nonpolar.

**Supplementary Table 3: Hydrogen bond frequencies for all MD simulations of LYRa3-hACE2**

| **LYRa3** | **hACE2** | **Sim1**  **(n=160)** | **Sim2**  **(n=168)** | **Sim3**  **(n=122)** | **Average** | **Std. error** |
| --- | --- | --- | --- | --- | --- | --- |
| G492 | K353 | 92.5 | 94.0 | 98.3 | 94.9 | 2.5 |
|  | G354 | 0.0 | 0.6 | 0.0 | 0.2 | 0.3 |
| N477 | Y83 | 89.3 | 98.2 | 90.1 | 92.5 | 4.0 |
|  | Q24 | 33.3 | 40.5 | 57.0 | 43.6 | 9.9 |
| T490 | D355 | 63.5 | 41.7 | 62.0 | 55.7 | 10.0 |
|  | Y41 | 10.7 | 25.6 | 1.7 | 12.6 | 9.9 |
|  | N330 | 0.6 | 4.8 | 0.0 | 1.8 | 2.1 |
| G486 | K353 | 16.4 | 73.2 | 53.7 | 47.8 | 23.6 |
| Y485 | K353 | 35.2 | 63.7 | 43.0 | 47.3 | 12.0 |
| R430 | E329 | 34.6 | 10.1 | 43.8 | 29.5 | 14.2 |
|  | Q325 | 1.9 | 0.6 | 0.0 | 0.8 | 0.8 |
| Y488 | Q42 | 22.6 | 19.6 | 16.5 | 19.6 | 2.5 |
|  | D38 | 0.0 | 26.2 | 3.3 | 9.8 | 11.6 |
| Y495 | E37 | 10.1 | 17.9 | 0.8 | 9.6 | 7.0 |
|  | R393 | 0.0 | 4.2 | 0.0 | 1.4 | 2.0 |
| Y479 | Q24 | 4.4 | 18.5 | 0.8 | 7.9 | 7.6 |
|  | Y83 | 0.0 | 0.6 | 0.0 | 0.2 | 0.3 |
| N483 | K31 | 9.4 | 2.4 | 9.1 | 7.0 | 3.2 |
|  | E35 | 0.6 | 0.6 | 4.1 | 1.8 | 1.7 |
| W480 | K31 | 10.1 | 0.0 | 9.1 | 6.4 | 4.5 |
| Y444 | H34 | 3.8 | 0.6 | 14.0 | 6.1 | 5.7 |
| N491 | K353 | 8.8 | 0.0 | 9.1 | 6.0 | 4.2 |
|  | Y41 | 4.4 | 5.4 | 0.0 | 3.3 | 2.3 |
| S446 | K31 | 1.3 | 0.0 | 6.6 | 2.6 | 2.9 |
| D467 | Q24 | 2.5 | 0.0 | 0.0 | 0.8 | 1.2 |
| L482 | K31 | 0.6 | 0.0 | 1.7 | 0.8 | 0.7 |
| S437 | Q42 | 0.0 | 1.2 | 0.0 | 0.4 | 0.6 |
| Q496 | Q325 | 0.6 | 0.0 | 0.0 | 0.2 | 0.3 |
|  | T324 | 0.0 | 0.6 | 0.0 | 0.2 | 0.3 |

Hydrogen bond frequencies (%) for three independent MD simulations of LYRa3 RBD bound to human receptor ACE2 (details in Methods). The number of sampled conformations is shown in parenthesis.

**Supplementary Table 4: Hydrogen bond frequencies for all MD simulations of Ty-HKU4-hDPP4**

| **Ty-HKU4** | **hDPP4** | **Sim1 (n=96)** | **Sim2 (n=171)** | **Sim3 (n=126)** | **Average** | **Std. error** |
| --- | --- | --- | --- | --- | --- | --- |
| E518 | Q344 | 93.8 | 96.5 | 95.2 | 95.2 | 1.1 |
|  | A291 | 20.8 | 53.8 | 59.5 | 44.7 | 17.1 |
| N514 | R317 | 92.7 | 95.9 | 94.4 | 94.4 | 1.3 |
|  | Y322 | 1.0 | 0.0 | 0.0 | 0.3 | 0.5 |
| K506 | A289 | 54.2 | 48.0 | 57.1 | 53.1 | 3.8 |
|  | T288 | 34.4 | 31.6 | 32.5 | 32.8 | 1.2 |
| K547 | I295 | 50.0 | 49.7 | 50.8 | 50.2 | 0.5 |
|  | L294 | 2.1 | 1.2 | 1.6 | 1.6 | 0.4 |
| E541 | K267 | 37.5 | 38.0 | 23.8 | 33.1 | 6.6 |
| N468 | R336 | 31.3 | 14.6 | 46.8 | 30.9 | 13.2 |
|  | G335 | 0.0 | 0.0 | 0.8 | 0.3 | 0.4 |
| D542 | K267 | 12.5 | 29.2 | 28.6 | 23.4 | 7.7 |
|  | Q286 | 6.3 | 0.6 | 0.0 | 2.3 | 2.8 |
| Q515 | R317 | 3.1 | 23.4 | 29.4 | 18.6 | 11.2 |
|  | S292 | 44.8 | 0.0 | 0.0 | 14.9 | 21.1 |
|  | Y322 | 0.0 | 2.3 | 0.8 | 1.0 | 1.0 |
| S465 | D331 | 0.0 | 2.3 | 51.6 | 18.0 | 23.8 |
|  | S333 | 4.2 | 19.3 | 19.8 | 14.4 | 7.3 |
|  | R336 | 0.0 | 12.3 | 0.0 | 4.1 | 5.8 |
|  | S334 | 1.0 | 0.0 | 0.8 | 0.6 | 0.4 |
| D516 | Y322 | 38.5 | 0.6 | 1.6 | 13.6 | 17.7 |
| Y460 | G335 | 8.3 | 4.7 | 2.4 | 5.1 | 2.5 |
|  | S333 | 0.0 | 0.0 | 3.2 | 1.1 | 1.5 |
|  | S334 | 3.1 | 0.0 | 0.0 | 1.0 | 1.5 |
|  | T283 | 0.0 | 1.2 | 0.0 | 0.4 | 0.6 |
| R462 | S333 | 0.0 | 11.7 | 0.0 | 3.9 | 5.5 |
|  | E332 | 0.0 | 1.2 | 0.0 | 0.4 | 0.6 |
| C590 | N42 | 8.3 | 0.0 | 0.0 | 2.8 | 3.9 |
| S505 | R336 | 0.0 | 0.0 | 7.9 | 2.6 | 3.7 |
| D587 | D216 | 6.3 | 0.0 | 0.0 | 2.1 | 2.9 |
|  | N40 | 4.2 | 0.0 | 0.0 | 1.4 | 2.0 |
| V580 | N109 | 4.2 | 0.0 | 0.0 | 1.4 | 2.0 |
| G543 | K267 | 0.0 | 0.6 | 1.6 | 0.7 | 0.7 |
| M592 | N44 | 2.1 | 0.0 | 0.0 | 0.7 | 1.0 |
| G585 | T213 | 2.1 | 0.0 | 0.0 | 0.7 | 1.0 |
|  | S217 | 2.1 | 0.0 | 0.0 | 0.7 | 1.0 |
| G464 | S333 | 0.0 | 0.0 | 1.6 | 0.5 | 0.7 |
| A466 | S334 | 0.0 | 0.0 | 1.6 | 0.5 | 0.7 |
| G467 | S334 | 0.0 | 0.6 | 0.8 | 0.5 | 0.3 |
| S579 | N109 | 1.0 | 0.0 | 0.0 | 0.3 | 0.5 |
| T584 | S217 | 1.0 | 0.0 | 0.0 | 0.3 | 0.5 |
| S459 | S334 | 1.0 | 0.0 | 0.0 | 0.3 | 0.5 |
| S588 | D216 | 1.0 | 0.0 | 0.0 | 0.3 | 0.5 |
| Y472 | R336 | 0.0 | 0.0 | 0.8 | 0.3 | 0.4 |

Hydrogen bond frequencies (%) for three independent MD simulations of Ty-HKU4 RBD bound to human receptor DPP4 (details in Methods).The number of sampled conformations is shown in parenthesis.

**Supplementary Table 5: Hydrogen bond frequencies for all MD simulations of Bt133-hDPP4**

| **Bt133** | **hDPP4** | **Sim1 (n=135)** | **Sim2 (n=106)** | **Sim3 (n=113)** | **Average** | **Std. error** |
| --- | --- | --- | --- | --- | --- | --- |
| E518 | Q344 | 97.0 | 100.0 | 100.0 | 99.0 | 1.4 |
|  | A291 | 51.1 | 16.0 | 14.2 | 27.1 | 17.0 |
| N514 | R317 | 96.3 | 95.3 | 95.6 | 95.7 | 0.4 |
|  | Y322 | 0.7 | 0.0 | 0.0 | 0.2 | 0.3 |
| Q515 | S292 | 88.1 | 74.5 | 50.4 | 71.0 | 15.6 |
|  | R317 | 5.9 | 0.0 | 0.9 | 2.3 | 2.6 |
|  | Y322 | 0.0 | 0.9 | 1.8 | 0.9 | 0.7 |
| N468 | R336 | 64.4 | 49.1 | 16.8 | 43.4 | 19.8 |
| K506 | A289 | 57.8 | 0.9 | 57.5 | 38.7 | 26.7 |
|  | T288 | 25.2 | 7.5 | 32.7 | 21.8 | 10.6 |
| K547 | I295 | 38.5 | 18.9 | 53.1 | 36.8 | 14.0 |
|  | L294 | 5.2 | 16.0 | 0.0 | 7.1 | 6.7 |
| E541 | K267 | 21.5 | 3.8 | 4.4 | 9.9 | 8.2 |
| D542 | K267 | 19.3 | 0.9 | 8.0 | 9.4 | 7.5 |
|  | Q286 | 2.2 | 9.4 | 8.8 | 6.8 | 3.3 |
| S465 | S334 | 7.4 | 7.5 | 0.0 | 5.0 | 3.5 |
|  | S333 | 0.0 | 0.0 | 6.2 | 2.1 | 2.9 |
| Y460 | G335 | 3.7 | 0.9 | 1.8 | 2.1 | 1.2 |
|  | S334 | 0.0 | 1.9 | 0.0 | 0.6 | 0.9 |
| S459 | S334 | 3.7 | 0.0 | 0.0 | 1.2 | 1.7 |
| C590 | N42 | 0.7 | 0.0 | 0.0 | 0.2 | 0.3 |
| D516 | Y322 | 0.7 | 0.0 | 1.8 | 0.8 | 0.7 |
| G585 | T213 | 0.0 | 0.9 | 0.0 | 0.3 | 0.4 |
|  | S217 | 0.0 | 0.9 | 0.0 | 0.3 | 0.4 |
| P591 | N42 | 0.0 | 0.9 | 0.0 | 0.3 | 0.4 |
| R462 | S333 | 0.0 | 0.0 | 7.1 | 2.4 | 3.3 |
| G543 | K267 | 0.0 | 0.0 | 1.8 | 0.6 | 0.8 |

Hydrogen bond frequencies (%) for three independent MD simulations of Bt133 RBD bound to human receptor DPP4 (details in Methods). The number of sampled conformations is shown in parenthesis.

**Supplementary Table 6: Alpha and beta coronaviruses used to create the phylogenetic tree in Supplementary Fig. 2**

| **Non-human Sarbecovirus (100)** |
| --- |
| MZ190138, MZ190137, MT799524, MT799523, MT799521, MT782114, MT072864, MT040336, MT040335, MT040334, MT040333, MN996532, MK211378, MK211377, MK211376, MK211374, MG772934, MG772933, LC556375, KY417152, KY417151, KY417150, KY417149, KY417148, KY417147, KY417146, KY417145, KY417144, KY417143, KY417142, KY352407, KU973692, KT444582, KP886809, KP886808, KJ473816, KJ473815, KJ473814, KJ473813, KJ473812, KJ473811, KF569997, KF569996, KF367457, KF294457, KC881006, KC881005, JX993988, JX993987, JX163927, JX163926, GQ153548, GQ153547, GQ153544, GQ153543, GQ153542, FJ959407, FJ588686, DQ648857, DQ648856, DQ514532, DQ514531, DQ514530, DQ514529, DQ514528, DQ412043, DQ412042, DQ084199, DQ071615, DQ022305, AY687372, AY687371, AY687370, AY687368, AY687365, AY687362, AY687361, AY687360, AY687359, AY687356, AY687355, AY687354, AY686863, AY627048, AY627047, AY627045, AY613952, AY613950, AY613948, AY572038, AY572037, AY572036, AY572034, AY545919, AY545915, AY515512, AY304489, AY304488, AY304486, NC_014470* |
| **Human coronavirus SARS-CoV (4)** |
| AY525636, AY278554, AY278489, AY274119* |
| **SARS-CoV-2 from human host (12)** |
| QOT58228, QOQ10156, QNO86975, QNO84047, QNO82103, QMU91735, QMJ19657, QMI94525, QKU52833, QKE51026, QJD47286, YP_009724390* |
| **SARS-CoV-2 from mink host (8)** |
| QNJ45226, QNJ45178, QNJ45142, QNJ45106, QJS39579, QJS39567, QJS39543, QJS39507 |
| **SARS-CoV-2 from feline host (6)** |
| QLC48479, QLC48467, QLC48455, QLC48443, QLC48419, QLC48407 |
| **hCoVOC43 from human host (8)** |
| QEG03794, AXX83375, AVR40342, ATP16767, AIV42005, AIV41987, AGT51561, YP_009555241* |
| **hCoVOC43 from non-human host (2)** |
| AWW13519, AWW13511 |
| **Human coronavirus HKU1 (6)** |
| AZS52618, ARB07438, AGW27872, ABD75601, ABD75513, YP_173238* |
| **Human coronavirus NL63 (5)** |
| QEG03731, AWK59931, AGT51380, AFD98827, YP_003767* |
| **Human coronavirus 229E (20)** |
| QJY77954, QEO75985, ATI09437, ARK08642, APD51507, APD51499, ALA50256, ALA50249, ALA50165, ALA50151, ALA50144, ALA50137, ALA50130, ALA50123, ALA50088, AGT21360, AFR79257, AFI49431, YP_009194639*, NP_073551* |
| **MERS from human host (9)** |
| QGW51920, QCQ29075, QBF80611, ASU45719, ANF29261, AKN24830, AKN24812, YP_009047204*, YP_007188579* |
| **MERS from non-human host in the Middle East (66)** |
| QOU08625, QOU08585, QOU08574, QOU08541, QOU08497, QCI31480, QCI31469, ASU91208, ASU91010, ASU90857, ASU90802, ASU90791, ASU90747, ASU90681, ASU90604, ASU90549, ASU90527, ASU90516, ASU90439, ASU90406, ASU90373, ASU90362, ASU90340, ASU90329, ASU90307, ASU90241, ASU90230, ASU90197, ASU90186, ASU90175, ASU90142, ASU90076, ASU90010, ASU89988, ASU89966, ASU89955, ASU89944, ASU45818, AQZ41296, ANI69922, ANI69900, ANI69889, ANI69878, ANI69835, ANI69824, ALL26409, ALL26396, ALA50067, ALA49957, ALA49803, ALA49671, ALA49660, ALA49649, ALA49473, ALA49462, ALA49451, ALA49396, ALA49374, ALA49363, ALA49352, ALA49341, AHY22565, AHY22555, AHY22545, AHX71946, AHX00711 |
| **MERS from non-human host in Africa (11)** |
| QGV13484, QBM11748, AXP07345, AVN89387, AVN89365, AVN89324, AUM60024, AUM60014, ATQ39390, AHY61337, AGY29650 |
| **Porcine epidemic diarrhea virus (11)** |
| QKV43727, QGQ60330, QGQ60306, QBO24651, AZL47192, AVI57403, AOC38021, AKH45338, AJW67223, AIR95864, NP_598310* |
| **Betacoronavirus 1 (24)** |
| AZU96327, AVV64341, AVN88332, ANJ04728, ANJ04717, ACT11019, ACJ66990, ACJ66977, ACJ66961, ACJ66946, ABP87990, ABP38313, ABP38306, QEY10657, QEY10649, QEY10641, QEY10633, QEY10625, AHN64783, AHN64774, ACT11030, ACJ35486, ABI93999, NP_150077* |
| **Other Betacoronaviruses (13)** |
| QDF43840, AYR18625, YP_009824982*, YP_009513010*, YP_009273005*, YP_009113025*, YP_009072440*, YP_005454245*, YP_003858584*, YP_003029848*, YP_001039971*, YP_001039962*, YP_001039953* |
| **Other Alphacoronaviruses (39)** |
| QLE11825, QKV43713, QER90704, QEH62669, QDF43790, AYR18493, AYR18455, AYR18412, AVY53336, AKV62755, AIA62271, AGZ84526, AFX81098, AFH55121, AFH55111, AEQ61968, ABG89317, YP_009824974*, YP_009824967*, YP_009755890*, YP_009389425*, YP_009380521*, YP_009336484*, YP_009328935*, YP_009256197*, YP_009201730*, YP_009200735*, YP_009199790*, YP_009199609*, YP_009199242*, YP_009019182*, YP_008439202*, YP_006908642*, YP_004070194*, YP_001718612*, YP_001718605*, YP_001552236*, YP_001351684*, NP_058424* |
| **Other coronaviruses (80)** |
| QOE77287, QOE77277, QOE77268, QNL24139, QNL24130, QNL24121, QKX95789, QKX95780, QKN89963, QKF94914, QJF53986, QJF53977, QID98969, QHA24724, QHA24718, QHA24710, QHA24703, QHA24696, QHA24687, QHA24678, QHA24671, QHA24665, QGA70702, QGA70692, QDE12097, QCX35167, QCX35160, QAS69029, AXY04083, AXP98439, AWH65932, AWH65921, AWH65910, AWH65899, AWH65888, AWH65877, AVP25406, AVM80466, ATN23889, ASR18946, ASR18938, ASL68953, ASL68941, ASL24654, APD51491, ALA50080, AIA62352, AIA62343, AIA62234, AIA62227, AIA62220, AIA62212, AIA62206, AIA62200, AFU92131, AFU92122, AFU92113, AFU92095, AFU92086, AFU92070, AFE48827, AFE48817, AFE48805, ADM33582, ADM33574, ADM33566, ADM33558, ADI80523, ACA52157, ABQ57224, ABQ57216, ABN10935, ABN10927, ABN10919, ABN10893, ABN10884, ABN10866, ABN10857, ABN10848, ABG47052 |

Accession numbers of the 424 alpha and beta coronaviruses used to create the phylogenetic tree in Supplementary Fig. 2. Reference sequences are marked with an asterisk. The total number of viruses for each cell is shown in parenthesis.
